# Illustrations of the Natural History of Worcestershire

**Published:** 1835-01-01

**Authors:** 


*Illustrations of the Natural History of Worcestershire.* By CHARLES HASTINGS, M.D.—London and Worcester, 1834. 8vo. pp. 184.

THIS book belongs to a genus, of which we should be glad to find the species rapidly increase. It is a prodromus of the natural history of Worcestershire, and we doubt not that it will be followed by the publication of much more satisfactory and ample details. As a first attempt, it is most creditable; and, from the additions which have been made to the lecture, as originally delivered, it has become not only a portly, but an important volume. Our libraries are often said to be very rich in county histories: we have consulted them, and too often we have found them lamentably poor. True it is that we have been overwhelmed with cumbrous folios, but very little county history do they contain. Their authors, instead of giving a history of the counties, have collected memoirs of the men born in them, and copied the memorials sculptured on their tombs. Our neighbours gave a memorable instance of their discrimination between places and people, when they changed the title of their monarch from "King of France" to "King of the French:" and, although to some this seemed an unnecessary refinement, still it is not a distinction without a difference: and of this difference we have now an illustration before us; for, while we are rich in the history of county-men and their works, we are poor in the extreme in the histories of our several counties. We have no objection to books devoted to the commemoration of the worthies of England, but we likewise desire some memorials of the counties themselves, beyond the scanty information usually given of their boundaries and superficial extent. The natural historian demands that researches be made into the geological structure of the country, the variations of its climate, whether produced by altitude or latitude, or affected by local peculiarities, as the vicinity of the ocean, the passage of rivers, the prevalence of marshes, and the abundance or destitution of wood: these, combined with inquiries concerning the plants and animals, whether indigenous or naturalized, will lead the way to investigations that more immediately, although not more powerfully, concern our race, viz. the productiveness of the soil, the salubrity of the air, the prevalence of disease, and the duration of life. Such are the proper materials for county histories; and if to these the chronologist may choose to add the annals of each place, or the antiquarian to subjoin copies of epitaphs, and other archæological treasures, we object not: we only demur to folios being filled with monumental inscriptions and obsolete local customs, to the exclusion or neglect of the natural condition of the county itself.

The foundation of provincial literary institutions and philosophical societies, would seem likely to fill up the hiatus in our literature indicated above; and, if the members of such institutions fairly perform their duties, much is in their power: they have the means of examining and investigating on the spot the several productions of their various localities, of collecting specimens in their museums, and publishing accounts in their Transactions; which museums and Transactions would then become the most valuable sources of reference to general writers. But we fear that these associations are misused, and their objects, as scientific institutions, very much perverted. The members too often have "itching ears;" they go to the meetings, rather to be amused than instructed,—rather to while away a vacant hour, than to lend that hour on usury for the advance of knowledge. The conductors are well aware of this feeling; or, if not at first fully sensible of it, they soon become convinced of its strength, by the falling-off in the subscriptions, if they fail to amuse the subscribers. The philosophers in and out of petticoats must be made to laugh, and then they are contented to be members of a learned society; but, if most of the meetings, or many of them, be devoted to really scientific subjects, the philosophers yawn, go to sleep, and withhold their guineas.

Let us, however, not be misunderstood. We object not to people being amused, and every liege subject of our gracious king is at full liberty to amuse himself as he himself thinks fit: but let him not call folly philosophy; let him not desecrate our altars;—we desire none of his society.

It is time, however, that we turn to the volume before us; and, however encouraging the *prospect* may be which opens with Dr. Hastings' Lecture, the retrospect is anything but pleasing. The Doctor takes shame to his native county for the backwardness there evinced in the pursuit of natural knowledge: we fear, however, that Worcestershire should not be made to stand alone on the stool of penance, though her sin be grievous.

"In this county the study has been so much neglected by the great mass of the community, that every thing is to be done. It is positively necessary that the student of, as well as the proficient in, natural history, should have books to which he can refer frequently, for without these he cannot advance himself, or know what has been done by others. With grief and shame be it spoken, that we are in this town lamentably deficient in this respect. The transactions even of the several societies that exist, and are actively employed in the advancement of knowledge, are not to be met with in our public libraries. The Transactions of the Linnæan and the Geological Societies, and of several others, we look for in vain; and we have also to deplore an entire neglect of all foreign works, which are so abundantly rich in natural history. It will in itself form a strong attraction to cultivated individuals to become members of our newly formed society, if they find that this great blank in the literature of our city is about to be filled up; which in the course of a few years it may be, by judicious measures being adopted." (P. 7.)

The Worcestershire Society appears to be founded on a very comprehensive, and, we think, satisfactory basis. Besides the formation of a library and museum, and the institution of courses of lectures, the members are associated in different sections, according to the peculiar tendency of their several pursuits; and the purpose of these sections is to collect and communicate specimens and observations to the society in general, and, through the society, to the public at large. The principal of these sections are the following: 1, Statistics; 2, Zoology; 3, Botany; 4, Geology and Mineralogy; 5, Meteorology.

These several sections form the theses for the several divisions of Dr. Hastings' discourse; and we shall conclude our notice of this volume by making a few extracts from them in succession.

During our rambles last summer, we had the pleasure of meeting Mr. Abraham, the inventor of the grinder's magnetic guard, an apparatus which we had always admired as a most philosophic application of magnetic power, and perhaps had admired the more, as it is one of the very few applications of magnetism to practical purposes; *i. e.* to aid the mechanic arts. But we were much disappointed to find that, even in Sheffield, the town celebrated for its invention, it is very little used. Dr. Hastings gives a parallel instance of its neglect at Redditch, where needle-making is carried on to a very great extent; and adds a quotation, illustrative of the infatuation of the people employed in this and such other deleterious trades.

"The excessive earnings of the needle-pointers at Redditch maintain a succession of men who taint the whole neighbourhood with profligacy, who, with the most perfect consciousness, devote themselves to an early death for the sake of a life of idleness and debauchery, and who acquire such a callous indifference to the fate which they know awaits them (premature death from the grinders' asthma, occasioned by the constant inhalation of an atmosphere loaded with metallic particles), that their apprehensions are said to have been excited lest Mr. Abraham's invention (Abraham's magnetic guard for collecting the steely particles,) should be successful enough to affect their wages." (P. 20.)

As an instance of the effect of strata upon health, we subjoin the following extract.

"It appears, from what I have before stated, that there is a great variety of soil in the county of Worcester, consisting of sand, bogs, debris of rock, lime, clay, and loam. Immediately under this soil, and the superficial beds of gravel and clay, the principal strata of the county are those of the red sandstone formation.

"Dr. Henry, of Manchester, in estimating the advantages of this kind of stratification upon health, says: 'The only principles on which the strata of any district lying beneath the soil and superficial beds of clay and gravel, appear capable of exerting an influence over the health of its inhabitants, are, as those strata absorb water more or less readily and completely, thereby affecting the hygrometrical state of the atmosphere; and as they furnish, by springs and rivers, water more or less impregnated with foreign ingredients, and therefore less or more fit for the use of man. Under the first view, the red sandstone is well adapted, by the avidity with which it imbibes water, to moderate the evils of a rainy climate like that of Lancashire. Under the second aspect, this rock furnishes an abundant supply of beautifully clear water, agreeable to the palate, but holding in solution much carbonate of lime, and a little sulphate of that earth, both of which are deposited on boiling. There is no reason to suppose that these impregnations have any effect unfavorable to health. They can have no tendency to produce calculous diseases, which were once imputed to them, but which have been shewn to be produced by causes quite independent of the qualities of water, and to depend on morbid operations of the animal economy. The almost universal freedom of the red sandstone from noxious metals (lead and copper being rarely found in it,) adapts it for the purpose of an excellent natural filter. By its spontaneous decomposition, also, this sandstone is known to furnish an excellent sandy loam, one of the most desirable that can be found for the production of every vegetable; and in this manner it cannot but materially contribute to the salubrity of any country of which it is the prevailing rock.'

"In confirmation of the truth of the principles here laid down by Dr. Henry, it may be stated that the whole of this county, in which the sand stone prevails, enjoys a great freedom from severe febrile diseases, the worst forms of typhus fever being rarely witnessed; and from my own experience I may assert that the hilly parts of Worcestershire, where there is a clay soil, as about Broadway, and the line of hills running in that direction, are much more prone to be affected with fever than the sandstone district which comprises the lower parts of the county. This immunity from severe forms of fever is not confined to the rural population; but is in a great measure also to be observed in the large towns. In Worcester it is a very rare thing to meet with very severe cases of fever, and the worst forms of the disease in this town are seen after flood-time, in the vicinity of the river Severn. This river, which rises in Plinlimmon, in Montgomeryshire, becomes of considerable size before it enters Worcestershire, a little above Bewdley. The bed, in its entire course through the county, is several feet below the banks; it has been stated, on an average eighteen feet. This circumstance necessarily detracts considerably from the effect that would otherwise arise from so fine a stream passing through so rich a country. It does not, however, prevent the valley through which the river passes being frequently inundated, particularly in the southern parts of the county, as about Upton, where, after floods, (which almost surround the town,) the population is liable to sore throat, bronchitis, and low fever.

"The stream of the Severn is much increased after entering the county by three small rivers and two canals, which join it before it reaches Worcester. The river flows rapidly by Worcester in a winding, but for the most part south-easterly direction. The chief streets of the city are situated several feet above the river, and the consequence is, that although the river rises very high in time of floods, the inhabitants of these parts are not affected by them; but those in the lower parts, chiefly inhabited by the poor, as in Turkey, Cripplegate, and Hylton lane, are very liable to be inundated; and thus, from the stagnation of water in the ditches and low grounds, miasmata arise, and the inhabitants, consequently, after rainy seasons, suffer from fever and inflammatory disorders.

"From want of draining, also, other parts of the city are rather liable to disease. This objection unfortunately applies to some of the houses that have been most recently erected: in the Blockhouse Fields, for example, the houses are erected upon a low marshy ground; and, from the circumstance of the bed of the canal, which runs near it, being higher than the foundation on which the houses are built, it is difficult to get an effective drainage; the result is, as the books of our charitable institutions shew, that low fever is more frequent here than in other parts. Does not such a fact as this, in conjunction with others of a similar nature, shew that it would be desirable that an act should pass the legislature to compel those who erect any considerable range of dwelling-houses so to choose their site that the necessary drainage may be effected; the neglect of which must necessarily produce disease, and its consequences, a squalid population. But, notwithstanding these defects in the medical police of Worcester, it must be conceded that fever is seldom epidemic in it. Nor is it only in the town of Worcester that we have the satisfaction to observe this comparative freedom from fever. Even in Kidderminster, where a large manufacturing population is pressed together, and where formerly, in the days of Dr. Johnstone, in the last century, scarlet fever and malignant sore throat, were so fearful and so destructive, these diseases now very seldom assume a severe form. The improvement in the health of the town in this respect may fairly in a great degree be attributed to a careful attention to the state of the small river, the Stour, which passes through it, by which accumulations of filth are prevented, and much of the ground that was formerly marshy is no longer visited by floods.

"Ague as a disease has almost ceased in this county; we rarely see a case of it: although, within the last few years, it has been rather more prevalent than formerly.

"By consulting our Infirmary records, I find that fifty years ago, a large proportion of cases admitted to that institution were for ague; but I doubt whether within the last twenty years the whole of the ague patients that have been treated at the Infirmary amount to so large a number as ten. This beneficial result may fairly be traced to improved cultivation of the soil." (P. 29.)

Dr. Hastings has collected a formidable list of aged individuals, to illustrate the salubrity of the county; as for example:

"In taking the account of the population of Droitwitch in 1821, there were found six sisters, all of them widows, whose united ages amounted to 443 years, viz. Elizabeth Everton, eighty-six; Ann Underwood, seventy-seven; Mary Bourne, seventy-five; Martha Blackford, seventy-one; Margaret Noake, sixty-eight; Hannah Kendall, sixty-six.

"The united ages of the sixteen men, inmates of St. Oswald's Hospital, in September, 1831, amounted to 1,123 years; and the twelve women, partakers of this excellent charity, had collectively attained the advanced age of 826 years.

"At Hawford, in the parish of Claines, December the 17th, 1792, lived Samuel Corbin, a member of the Society of Friends. He had two brothers and two sisters, all of whom attained a great age.

| | | | | |
|---|---|---|---|---|
| John Corbyn, | was born in | 1700, | and died in | 1785. |
| Samuel Corbyn, | — | 1706, | — | 1799. |
| Thomas Corbyn, | — | 1711, | — | 1791. |
| Hannah Palmer, | — | 1713, | were living in 1796. | |
| Candia Burlingham, | — | 1714, | | |

The father died aged eighty-four, and the mother aged ninety-seven.

"There is now living in the parish of Stone, in this county, a poor man, aged 104, who spent his early days at manual labour, and now enjoys a peaceful and serene old age." (P. 37.)

"Mr. Locke mentions, in his journal for 1681, that in that year he saw a woman, named Alice George, who was then 108 years of age, who was born at Saltwych, in Worcestershire; her father lived to eighty-three, her mother to ninety-six, her mother's mother to 111; 'she never,' adds Mr. Locke, 'took any physic but once, about forty years since.'" (P. 38.)

As we have given an instance of an old lady who lived upwards of a century, and only took one dose of physic, we will close this account with that of a gentleman, who is said to have been worried to death at the immature age of eighty-eight.

"In Arely church, near Stourport, is the following curious inscription: 'Here lyeth the body of William Walsh, gentleman, who died the third day of November, 1702, aged eighty-eight, son of Michael Walsh, of Great Shelsley, who left him an estate in Shelsley, Hartlebury, and Arely; who was ruined and undone by three quakers and three lawyers, and a fanatick to help them out. (P. 42.)

It may be interesting to some of our zoological friends to know that the old English black rat still maintains a station in Worcestershire.

"Two species of rats are found, *Mus rattus* and *M. decumanus.* The latter species is very numerous, and proves a serious annoyance in old houses. The black rat, *M. rattus*, the original English species, has recently much engaged the attention of naturalists, and particularly of one of our own members, Mr. Jabez Allies, from the curious fact, that, like the Indians in the New World, the race has almost dwindled away before the more ferocious and sagacious brown Norway rat, *M. decumanus.* Whether the popular opinion that the blacks are destroyed by the brown ones, or Dr. Fleming's suggestion that the substitution of tiled and slated roofs for thatch, is the correct cause of their declension, certain it is that, within the memory of individuals now living, the black rats which were formerly known to abound are now rarely met with, while the brown rats swarm everywhere. Though at some few farm-houses the black rats are found in company with the brown ones, yet it is believed that Mr. Dowding's, of Wick, is the only place in our vicinity where the black rat is found alone." (P. 61.)

The following fact deserves investigation.

"It is a curious circumstance, that though the salmon ascend many other tributaries of the Severn; and are frequently caught in the Teme, yet neither that fish, nor the shad, lamprey, nor lampern, ever attempt to enter the Avon at Tewkesbury, which joins the Severn at that place. There are certainly various impediments to the passage in mills and weirs, but such obstacles are in other places surmounted by salmon. It is however stated by the fishermen, that the salmon manifest the utmost aversion to the Avon water, and if forced into it by them, when deposited in the trunks of their boats, they turn round to escape, and soon die, if they are not relieved. It seems probable that some unpleasant vegetable particles are held in solution by the waters of the Avon, which, notwithstanding its universal praises by the poets, is in effect little better than a winding stagnant pool, and offers no advantages to the fish, who prefer a quick flowing stream with a gravelly bed, and dislike the muddy bottom of the Avon." (P. 77.)

From the lists given of native plants, the botany of Worcestershire would appear to be tolerably rich, but these catalogues do not afford any passages for extract. We confess that we are rather disappointed with this division of the lecture; for we know not any more sensible indicators of climate thau plants, and we should have been much pleased to have found that Dr. Hastings had turned his attention to this subject. In the geological and mineralogical sections, we have furthat has been often made, for want of a knowledge of the first principles of these interesting sciences.

"It is indeed but a few years ago, so complete was the ignorance of persons upon these subjects, that a shaft was sunk to raise gold from the hills of Malvern, and the individuals concerned lost a large property in the speculation. Setting aside, then, all considerations of the suitableness of such pursuits to our intellectual gratification, the mere desire of thrift should prompt us to become acquainted with the geological peculiarities of our county." (P. 89.)

The mildness of the vale of the Severn is well known, but the want of extended and accurate observation prevents any general statement of the climate of the county being as yet adventured; but, from some observations made at Woodthorpe, near Sheffield, and at Barbourne, near Worcester, the difference would appear to be much greater than could have been anticipated. We subjoin an extract, made by Dr. Hastings, as a specimen.

"Mean temperature of the last week in October, at twelve o'clock in the day:

| Barbourne. | Woodthorpe, |
|---|---|
| 60° 3′ | 55° 5′ |
| Mean of the month of November. | |
| 49° 16 | 44° 2′ |
| Mean of first week in December. | |
| 50° | 44° 4′ |
| Mean of the whole six weeks. | |
| 52° | 45° 32. |

(P. 126.)

We now take leave of Dr. Hastings, whose work affords us a double pleasure: first, from its intrinsic excellence; and, secondly, from its being an additional instance of the compatibility of the most ardent cultivation of science with the active pursuits of a professional life.

